# Stem cell-derived exosomes: emerging therapeutic opportunities for wound healing

**DOI:** 10.1186/s13287-023-03345-0

**Published:** 2023-04-26

**Authors:** Chuchao Zhou, Boyu Zhang, Yanqing Yang, Qiong Jiang, Tianyu Li, Jun Gong, Hongbo Tang, Qi Zhang

**Affiliations:** 1 Department of Plastic Surgery, Wuhan Third Hospital (Tongren Hospital of Wuhan University), Wuhan, 430060 China; 2 Department of Plastic and Cosmetic Surgery, Tongji Hospital, Tongji Medical College, Huazhong University of Science and Technology, 1095 Jiefang Avenue, Wuhan, 430030 Hubei China; 3 Department of Pharmacy, Xianning Central Hospital, The First Affiliated Hospital of Hubei University of Science and Technology, Xianning, 437000 Hubei China; 4 Trauma Center/Department of Emergency and Traumatic Surgery, Tongji Hospital, Tongji Medical College, Huazhong University of Science and Technology, Wuhan, China; 5 Department of Biliary-Pancreatic Surgery, Tongji Hospital, Tongji Medical College, Huazhong University of Science and Technology, 1095 Jiefang Avenue, Wuhan, 430030 Hubei China

**Keywords:** Wound healing, Stem cells, Exosomes, Regeneration, miRNA, lncRNA

## Abstract

Wound healing is a dynamic and highly sequential process involving a series of overlapping spatial and temporal phases, including hemostasis, inflammation, proliferation, and tissue remodeling. Mesenchymal stem cells (MSCs) are multipotent stem cells with self-renewal, multidirectional differentiation potential, and paracrine regulation. Exosomes are subcellular vesicular components 30–150 nm in size and are novel carriers of intercellular communication in regulating the biological behaviors of skin cells. Compared to MSCs, MSC-derived exosomes (MSC-exos) possess lower immunogenicity, easy storage, and highly effective biological activity. MSC-exos, mainly derived from adipose-derived stem cells (ADSCs), bone marrow-derived MSCs (BMSCs), human umbilical cord MSCs (hUC-MSCs), and other stem cell types, play a role in shaping the activity of fibroblasts, keratinocytes, immune cells, and endothelial cells in diabetic wounds, inflammatory wound repair, and even wound-related keloid formation. Therefore, this study focuses on the specific roles and mechanisms of different MSC-exos in wound healing, as well as the current limitations and various perspectives. Deciphering the biological properties of MSC-exos is crucial to providing a promising cell-free therapeutic tool for wound healing and cutaneous regeneration.

**Graphical Abstract:**

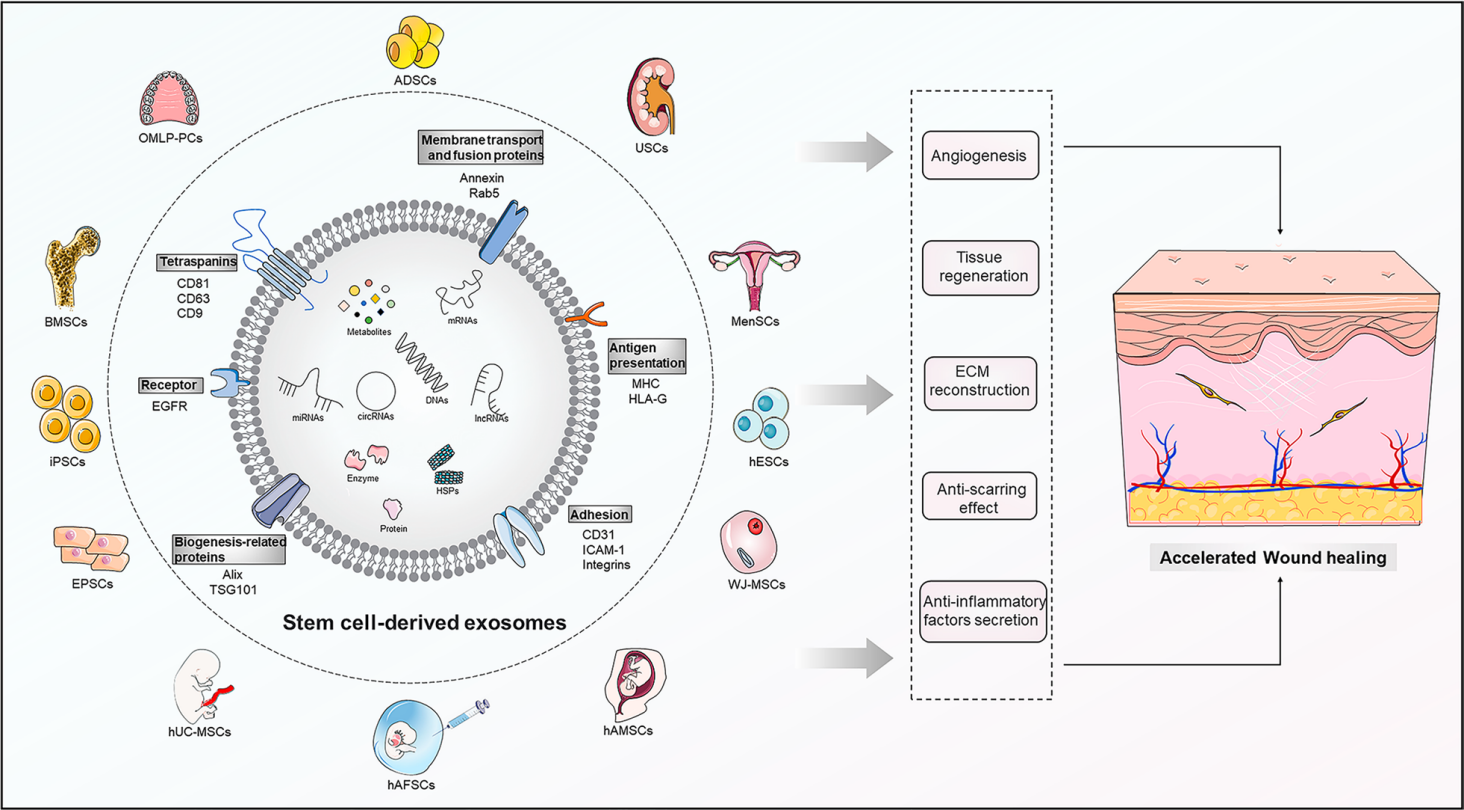

## Introduction

**Fig. 1:**
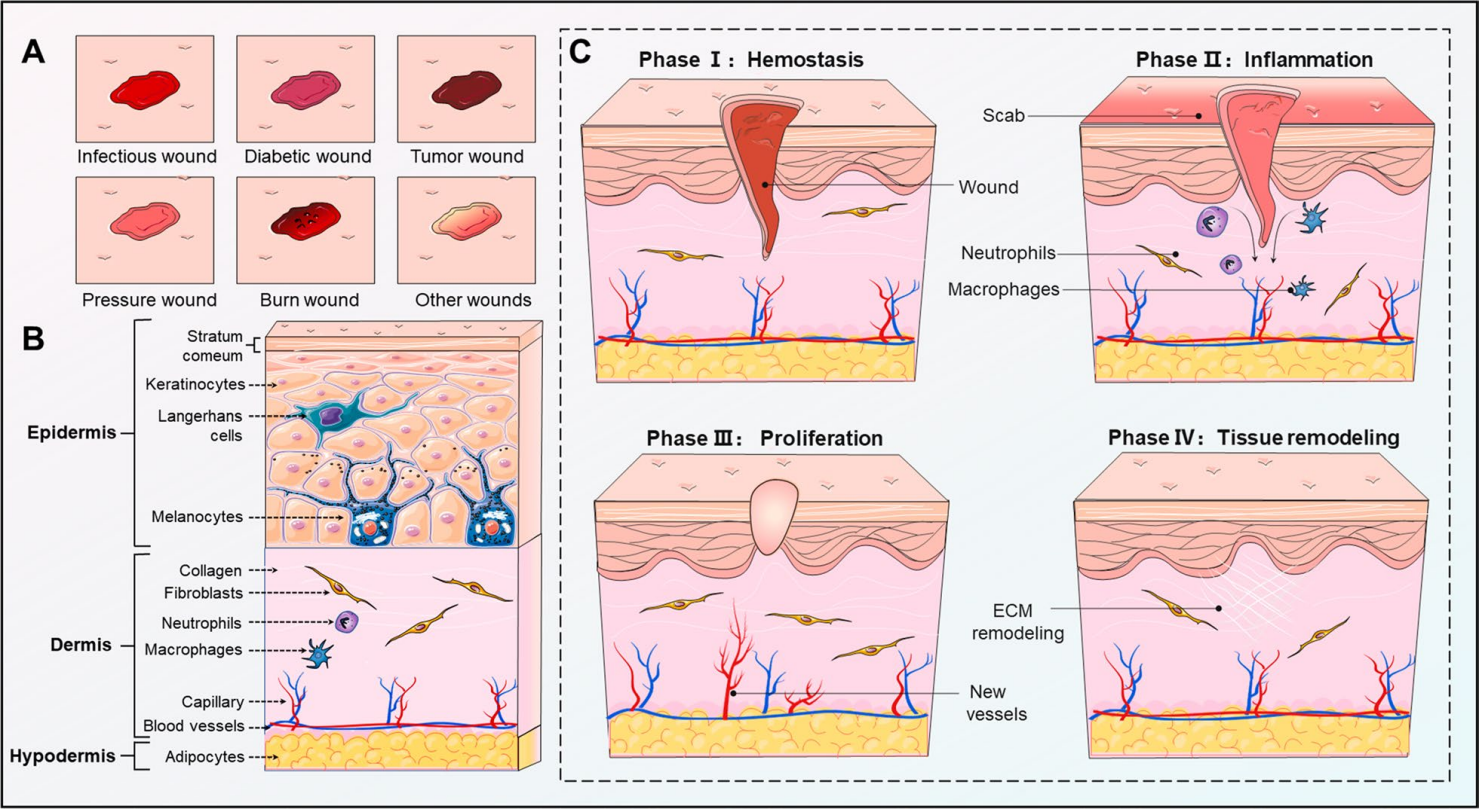
Wound types, skin structure, and wound healing phases. **a** Different wound types, mainly including infectious, diabetic, tumor, pressure, burn, and other wounds, such as trauma, radiation, drug-induced, ischemia and venous congestion wounds. **b** The skin structure consists of three layers, the epidermis, dermis, and hypodermis. The epidermis is primarily composed of keratinocytes, melanocytes, and Langerhans cells, contributing to a natural barrier against foreign objects. The dermis is a region rich in fibroblasts and immune cells, as well as collagen fibers, elastic fibers, reticular fibers, nerve endings, and capillaries, for maintaining the density and tightness of skin structure. The underlying hypodermis consists of adipocytes and connective tissue, which is conducive to warmth, cushioning, and reduction of mechanical stress. **c** The phase of wound healing is temporally overlapping and interdependent, including hemostasis, inflammation, proliferation, and tissue remodeling. Fibroblasts, keratinocytes, and immune cells are important skin cells in wound healing phases. In this figure was partly generated using Servier Medical Art (https://smart.servier.com), provided by Servier, licensed under a Creative Commons Attribution 3.0 unported license (https://creativecommons.org/licenses/by/3.0/)

Skin wounds are caused by various intrinsic pathological and external mechanical factors that damage the structure or integrity of skin tissues [1]. Skin wounds can lead to a series of local and even systemic physiological and pathological changes, which result in a heavy burden on patients and society. Wound healing is a highly sequential process of skin barrier function restoration and consists of temporally overlapping and interdependent phases, including hemostasis, inflammation, proliferation, and tissue remodeling [2]. During these phases, there are dynamic interactions between numerous different types of skin cells and immune cells that function at specific stages to reshape the wound healing process [3] **(**Fig. 1**)**. Currently, clinically conventional approaches, including various types of wound dressings, negative pressure suction, tissue engineering substitutes, and autologous skin grafts, are being employed to foster wound healing, but all have certain values and unsatisfactory points [4–6].

Mesenchymal stem cells (MSCs) are multipotent stem cells with self-renewal capability, multidirectional differentiation potential, and paracrine regulation [7]. Due to their easy isolation, in vitro expansion, and multipotent stemness, MSCs have been acknowledged as an important source of stem cells in the field of regenerative medicine, including tissue repair [8, 9]. In addition to MSCs in bone marrow, MSCs are also present in adipose, muscle, umbilical cord, several organs, and other tissues. Stem cells have excellent therapeutic effects in promoting skin remodeling, tissue vascularization, soft tissue regeneration, bone and cartilage repair, multi-tissue rejuvenation, and hair follicle regeneration. Presently, stem cells are primarily involved in facilitating skin wound healing through the paracrine function of multiple factors. Of note, the therapeutic utilization of stem cells in wound healing is also limited by storage challenges, mutation-related tumorigenicity, optimal cell activity, immune rejection, and ethical factors [10].

Exosomes are naturally occurring subcellular vesicular components 30–150 nm in size that can be secreted by almost all cells [11]. Exosomes are widely dispersed in body fluids, such as serum, saliva, milk, cerebrospinal fluid, urine, and semen [12]. Exosomes perform biological functions such as transmitting information, removing intracellular components, and acting as drug carriers by transporting a wide range of biologically active components that regulate physiological and pathological processes [13, 14]. Notably, stem cell exosomes possess unique biological functions similar to stem cells, as exosomes are the products and mappings of their parental cells. Stem cell exosomes are pivotal mediators of the biological effects of paracrine factors from stem cells, providing an ideal approach for further cell-free therapeutic wound healing [15]. MSC-derived exosomes (MSC-exos) also contain cytokines such as vascular endothelial growth factor (VEGF), transforming growth factor-β1 (TGF-β1), interleukin-6 (IL-6), interleukin-10 (IL-10), and hepatocyte growth factor (HGF), which facilitate angiogenesis and immunomodulation [16]. The main packaging components of MSC-exos, including metabolites, proteins, DNA, and non-coding RNAs (ncRNAs), can be internalized by recipient cells, such as fibroblasts, keratin-forming cells, immune cells, and endothelial cells (ECs), and further promote better wound repair by improving wound circulation, promoting wound vascularization, modulating inflammatory stress states, and recruiting stem cells [17]. In addition, the repair efficacy of MSC-exos can be improved by targeted editing of exosome content, pretreatment of MSCs, or artificial modification of exosome surface molecular receptors.

**Fig. 2:**
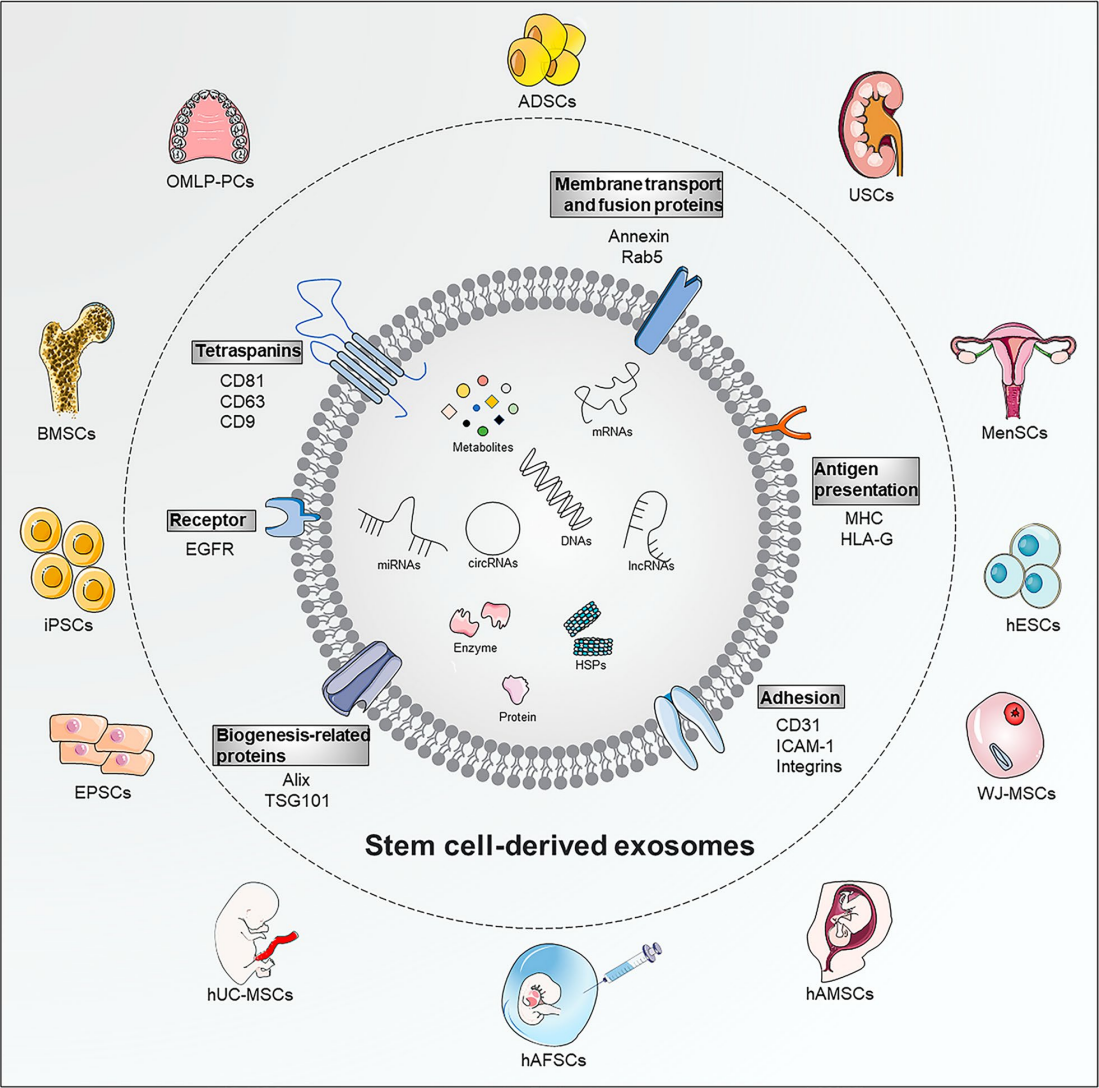
The compositions, biomarkers, and source of MSC-exos. Compared to normal exosomes, MSC-exos display similar compositions and biomarkers, which are related to biogenesis-related proteins (Alix, TSG101), membrane transport and fusion proteins (Annexin, Rab5), tetraspanins (CD81, CD63, CD9), specific receptor (EGFR), and antigen presentation (MHC, HLA-G). Various components, mainly metabolites, proteins, enzymes, HSPs, DNAs, mRNAs, miRNAs, lncRNAs, and circRNAs, are enclosed within MSC-exos. Multiple stem cell exosomes have good potential in wound healing, including ADSCs, BMSCs, hUC-MSCs, iPSCs, EPSCs, USCs, OMLP-PCs, MenSCs, WJ-MSCs, hAMSCs, hAFSCs, and hESCs. EGFR, epidermal growth factor receptor; MHC, major histocompatibility complex class; HLA-G, human leukocyte antigen-G; HSPs, heat shock proteins; mRNAs, messenger RNAs; miRNAs, microRNA; lncRNAs, long non-coding RNAs; circRNAs, circular RNAs. In this figure was partly generated using Servier Medical Art (https://smart.servier.com), provided by Servier, licensed under a Creative Commons Attribution 3.0 unported license (https://creastivecommons.org/licenses/by/3.0/)

In summary, MSC-exos potentially possess specific application advantages over MSCs in promoting wound healing. Adipose-derived stem cells (ADSCs), bone marrow-derived MSCs (BMSCs), and human umbilical cord MSCs (hUC-MSCs) are the most frequently reported exosome-producing cells used for wound healing **(**Fig. 2**)**. Therefore, this study focused on the specific roles and mechanisms of various MSC-exos in wound healing, demonstrating promising cell-free therapeutic strategies for wound healing and cutaneous regeneration.

## ADSCs

ADSCs are adipose tissue-derived MSCs that show self-renewal and multidirectional osteogenic, chondrogenic, and adipogenic differentiation [18]. ADSCs also possess the advantage of rich and wide sources, easy separation processes, low immunogenicity, and wide applications. Cell-free derivatives of ADSCs, including conditioned media, exosomes, and other active substances, induce paracrine effects and have been proposed as alternatives to traditional ADSC therapy [19]. In particular, due to their roles in regulating oxidative stress, immune cell infiltration, peri-wound vascularity, and inflammatory factor secretion in the wound microenvironment, ADSC-derived exosomes (ADSC-exos) are preeminent candidates for alleviating wound healing [20].

### ADSC-exos

ADSC-exos display similar effects to ADSCs in promoting wound regeneration and reducing keloid formation, with a much lower risk of immune rejection. Zhou et al. compared the efficacy of combined administration of ADSCs/ADSC-exos or single administration [21]. Combined administration tended to induce wound healing with better re-epithelialization, newly formed vessels, and fewer scar extensions. This study sheds new light on ADSC-exos and ADSC/ADSC-exos in wounds with attenuated scar formation. In addition, in vitro, ADSC-exos could be internalized by fibroblasts and increase N-cadherin, cyclin-1, proliferating cell nuclear antigen (PCNA), and collagen I/III in recipients [22]. Furthermore, ADSC-exos were recruited to wound sites to induce collagen I/III formation in the early healing period and prevented collagen deposition in the late period, thus benefiting scar-free wound healing. Chen et al. revealed that ADSC-exos could increase the proportions of collagen I/collagen III, TGF-β3/TGF-β1, and MMP3/TIMP1 by activating ERK/MAPK pathway, thus preventing fibroblasts from differentiating into myofibroblasts [23]. These factors synergistically promote the reconstruction of extracellular matrix (ECM) and potentially reduce scar formation in wound regeneration.

In a co-culture model, ADSC-exo stimulation improved the mobility and activity of fibroblasts with elevated expression levels of type I/III collagen, MMP1, bFGF, and TGF-β1 [24]. ADSC-exos mediated this function by increasing the p-AKT/AKT level to improve the healing rate. Zhang et al. also showed that ADSC-exos prominently promoted the activity of HaCaT cells by activating the AKT/HIF-1α pathway, consequently leading to accelerated healing [25]. In their further study, ADSC-exos reduced reactive oxygen species (ROS) production in human umbilical vein ECs (HUVECs) and improved mitochondrial function in a high-glucose environment [26]. The in-depth investigation confirmed that ADSC-exos could facilitate diabetic wound healing by modulating SIRT3/SOD2 to improve high-glucose-induced oxidative stress levels, promote angiogenesis, and reduce mitochondrial functional impairment and the inflammatory response. In full-thickness wounds in diabetic mice, ADSC-exos separated from diabetic mice could promote healing by enhancing dermal cell proliferation, keratinocyte (KC) proliferation, and angiogenesis [27]. Moreover, these ADSC-exos stimulated monocytes/macrophages to produce large amounts of TGF-β1 and activated the TGF-β/Smad3 pathway to stimulate fibroblast proliferation and activation.

### NcRNA from ADSC-exos

#### MiRNA from ADSC-exos

MicroRNAs (miRNAs) are a series of highly conserved small ncRNA molecules that can negatively regulate targeted miRNA expression via interference with transcriptional, translational, or epigenetic processes [28]. Through tissue-specific regulation of miRNAs, it is possible to remodel molecularly heterogeneous skin cells and help overcoming the challenges of wound regeneration.

Gene therapies represented by miRNA interference are susceptible to degradation in the wound microenvironment. Stem cell-based therapies and emerging targeted vector transport of exosomes are effective strategies for wound healing. Based on this, Lv et al. manufactured ADSC-exos to overexpress miR-21-5p, which could promote diabetic wound healing through re-epithelialization, collagen remodeling, angiogenesis, and vascular maturation [29]. MiRNA-146a-modified ADSC-exos promoted the mobility and proliferation of fibroblasts with up-regulation of SERPINH1 and p-ERK and neovascularization, resulting in ameliorated healing [30]. Zheng et al. indicated that miR-378 was abundant in ADSC-exos and could decrease oxidative stress injury in HaCaT cells by targeting caspase-3 [31]. Exosomal miRNA-125a-3p promoted the viability and migration of HUVECs and enhanced wound healing and angiogenesis in mice [32]. These functions were both probably mediated by miRNA-125a-3p-induced PTEN inhibition.

Phosphoinositide-3 kinases (PI3K) constitute a diverse family of kinases that govern a vast multitude of biological processes in mammalian cells, such as cell growth, proliferation, and viability [33]. The PI3K/AKT pathway is involved in skin homeostasis and the healing process. Based on high-throughput sequencing, Wang et al. reported that hypoxic ADSC-exos had different miRNA expression levels than ADSC-exos [34]. Specifically, up-regulated miR-21-3p/miR-126-5p/miR-31-5p and down-regulated miR-99b/miR-146-a in ADSC-exos promoted diabetic wounds and suppressed inflammatory factors via the PI3K/AKT signaling pathway. ADSC-derived exosomal miR-126-3p overexpression increased the wound healing rate, collagen deposition, and angiogenesis by inhibiting PIK3R2 in rat skin defects [35]. MiR-21 was highly expressed in ADSC-exos and could promote wound regeneration. MiR-21 could promote MMP-9 expression and inhibit TIMP-2 by activating the PI3K/AKT pathway in HaCaT cells [36].

#### LncRNA from ADSC-exos

Long non-coding RNAs (lncRNAs) are important heterogeneous RNAs with a length of more than 200 bp and cannot encode proteins. The functions of lncRNAs are mainly involved in modulating gene methylation and transcriptional activation and affecting translational progression and other processes [37]. Exosomal lncRNAs in ADSCs can be transferred to skin component cells to mediate the modifications of target genes and protein expression levels.

Endothelial progenitor cells (EPCs) in diabetic patients exhibited inhibited proliferation, migration, and angiogenesis and reduced Twist1 protein expression [38]. In a study by Qiu et al., exosomes from linc00511-overexpressing ADSCs restored the biological capacity of EPCs and accelerated angiogenesis by inhibiting PAQR3-induced degradation of Twist1 ubiquitination, thereby promoting diabetic foot ulcer (DFU) healing. LncRNA H19 in ADSC-exos could up-regulate SOX9 expression via miR-19b to activate the Wnt/β-catenin pathway, leading to accelerated proliferation, migration, and invasion of human skin fibroblasts (HSFs) and accelerated wound healing in skin tissues [39]. This study provided a novel perspective on the role of the lncRNA H19/miR-19b/SOX9 axis in the healing capability of ADSC-exos. ADSC-exosome-loaded XIST promoted wound healing through XIST/miR-96-5p/DDR2 [40]. Either miR-96-5p silencing or DDR2 overexpression enhanced the activating role of ADSC-exos.

MALAT1 is a frequently reported lncRNA that has attracted attention for its potential roles in angiogenesis and cellular activity [41]. Cooper et al. found that MALAT1 was enriched in ADSC-exos and could stimulate the human dermal fibroblasts (HDFs) migration and angiogenesis in ischemic wound closure in rats [42]. In addition, He et al. indicated that MALAT1 in ADSC-exos decreased the apoptosis of HaCaT cells and HDFs treated with H2O2 [43]. MALAT1-containing ADSC-exos activated the Wnt/β-catenin pathway by binding miR-124 to promote cutaneous wound healing. Additionally, exosomal lncRNA MALAT1 from ADSC-exos promoted the proliferation and migration of HSFs and was conducive to wound healing by manipulating the miR-378a/FGF2 axis [44]. These studies indicated the potency of MALAT1 in treating cutaneous wounds.

#### CircRNA from ADSC-exos

Circular RNAs (circRNAs) are ubiquitous endogenous ncRNAs that are covalently generated by backsplice connections to form single-chain circular molecules [45]. CircRNAs are important orchestrators in different wound healing stages with spatiotemporally altered signatures. However, exosomal circRNA has not been extensively investigated. Shi et al. found that mmu_circ_0000250 originating from ADSC-exos enhanced SIRT1 expression by miR-128-3p absorption and inhibited apoptosis via autophagic activation [46]. This function triggered diabetic wound healing in an accelerated process, emphasizing the therapeutic potential of the mmu_circ_0000250/miR-128-3p/SIRT1 axis. Exosomes from mmu_circ_0001052-modified ADSCs promoted angiogenesis in DFU via the miR-106a-5p and FGF4/p38MAPK pathways, which are essential for wound healing in DFU [47].

### Protein from ADSC-exos

ADSC-exos could prevent glucose-induced EPC senescence in vitro. Exosomes from ADSCs overexpressing Nrf2 enhanced this positive effect and facilitated DFU wound healing and vascularization by suppressing ROS and inflammatory cytokine expression [48]. Sun et al. explored whether EGR-1 in ADSC-exos could bind to the promoter of lncRNA-SENCR, which distinctively interacted with DKC1 to increase VEGF-A expression [49]. This study deciphered the role of ADSC-exos in promoting wound healing via the EGR-1/lncRNA-SENCR/DKC1/VEGF-A axis.

## BMSCs

BMSCs are multilineage progenitors with self-renewal, multidirectional differentiation, and pleiotropic paracrine functions [50]. BMSCs can repair damaged tissues and are employed in many different regenerative therapies. Purified BMSC-derived exosomes (BMSC-exos) have more specific distinct benefits in damaged tissue repair than BMSCs themselves, including superior stability, tissue permeability, excellent biocompatibility, and immunomodulatory properties.

### BMSC-exos

BMSC-exos are another promising source for cell-free skin regeneration. For instance, human BMSC-exos could suppress the TGF-β/Smad pathway to induce the proliferation of HaCaT cells and HDFs and accordingly enhance the wound healing process [51]. Specific gene editing and intervention preconditioning of BMSCs and BMSC-exos can enhance their effectiveness in wound healing. Specific drug-pretreated BMSC-exos are usually endowed with more vigorous cytokine secretion and cellular regulatory functionality.

### Pretreated BMSC-exos

Deferoxamine-pretreated BMSC-exos (DFO-exos) significantly promoted angiogenesis and diabetic rat wound healing [52]. In vitro, DFO-exos activated the PI3K/AKT pathway via miR-126-mediated PTEN down-regulation, thus enhancing angiogenesis. Yu et al. found that atorvastatin (ATV)-pretreated BMSC-exos (ATV-exos) facilitated angiogenesis and wound closure in a diabetic rat model [53]. In vitro, ATV-exos stimulated the activation of the AKT/eNOS pathway via miR-221-3p up-regulation, thus promoting EC angiogenesis. Hu et al. found that BMSC-exos pretreated with pioglitazone (PGZ-exos) obviously facilitated HUVEC viability and proliferation under high-glucose (HG) conditions in vitro and promoted collagen deposition and ECM remodeling with enhanced vascularization in a diabetic rat model [54]. In particular, PGZ-exos strengthened angiogenesis by activating the PI3K/AKT/eNOS pathway. Liu et al. found that melatonin-pretreated exosomes (MT-exos) apparently promoted diabetic wound healing [55]. Furthermore, MT-exos significantly suppressed the proinflammatory factors IL-1β, TNF-α, and iNOS but promoted the anti-inflammatory factors IL-10 and Arg-1. In addition, MT-exos increased the ratio of M2/M1 polarization by enhancing the PTEN/AKT pathway to modulate cytokine secretion.

### NcRNA from BMSC-exos

#### MiRNA from BMSC-exos

In a skin lesion model of HaCaT cells with H_2_O_2_ exposure, Shen et al. showed that BMSC-exos were able to strengthen skin wound healing partially by targeting the miR-93-3p/APAF1 axis. In addition, miR-93-3p knockdown in BMSC-exos antagonized this protective effect [56]. Liu et al. found that miR-155-inhibitor-loaded BMSC-exos enhanced KC migration, FGF-7 recovery, and anti-inflammatory effects in vitro and could also be utilized to treat a diabetic wound model by promoting collagen deposition, angiogenesis, and re-epithelization [57]. MiR-155-inhibitor and BMSC-exos had functional coordination in enhancing diabetic wound healing. Zhang et al. established and obtained exosomes originating from miRNA-126-overexpressing BMSCs and manipulated them to improve newly formed capillaries and wound healing [58]. Mechanistically, exosomal miRNA-126 promoted HUVEC angiogenesis by targeting the PIK3R2/PI3K/AKT signaling pathway, accompanied by elevated VEGF and Ang-1 expression levels. BMSCs could induce macrophage differentiation toward the M2 phenotype in vitro, and this immunomodulatory mechanism was important for wound healing. He et al. showed that miR-223 in BMSC-exos could modulate the M2 polarization phenotype and accelerate wound healing by targeting PKNOX1 [59].

#### LncRNA from BMSC-exos

The exosomal lncRNA KLF3-AS1 from BMSCs enhanced the expression of VEGF-A by down-regulating its target miR-383, thus inducing angiogenesis and promoting diabetic skin wound healing [60]. LncRNA HOTAIR was overexpressed in BMSCs and significantly accelerated angiogenesis and diabetic wound repair in db/db mice [61]. In the DFU model, BMSC-derived exosomal lncRNA H19 could be transmitted to fibroblasts, thus enhancing fibroblast proliferation and migration and inhibiting fibroblast apoptosis and inflammation [62]. This process was achieved by inhibiting the miR-152-3p/PTEN axis.

## hUC-MSCs

### hUC-MSC-exos

hUC-MSCs are typical self-renewing and multipotential adult stem cells that can differentiate into terminal cells. hUC-MSCs are popular because of their low immunogenicity, immunomodulation, ncRNA regulation, noninvasive harvesting process, easy in vitro expansion, and ethical compliance [63]. hUC-MSC-derived exosomes (hUC-MSC-exos) alleviated oxidative stress injury in HUVECs in vitro and strengthened vascular remodeling in vivo to improve diabetic wounds [64].

Previous studies have shown that denervated skin contributes to adverse wound healing consequences. Zhu et al. noted that hUC-MSC-exos could stimulate dermal fibroblasts to secrete nerve growth factors (NGFs) in vitro [65]. In a mouse model, hUC-MSC-exos could promote skin regeneration and full-thickness cutaneous wound healing by recruiting fibroblasts, stimulating their production of NGFs, and thereby promoting cutaneous nerve regeneration.

### MiRNA from hUC-MSC-exos

The function of hUC-MSCs in accelerating wound healing was partially mediated by exosomes. In particular, exosomal miRNAs can be delivered to skin recipient cells and promote downstream cascade signaling changes. Exosomes from hUC-MSCs could be absorbed by ECs and accelerated skin injury model. Furthermore, hUC-MSCs under hypoxic conditions presented enhanced miR-125b expression to inhibit p53 protein expression to alleviate hypoxia-induced cell apoptosis [66]. This study identified a reciprocal action between hUC-MSCs and ECs by exosomal miR-125b/TP53INP1 signaling in the hypoxic cutaneous microenvironment. Yang et al. discovered that 455-nm blue light exposure was an effective enhancer to strengthen the proangiogenic ability of hUC-MSC-exos [67]. This phenomenon was achieved partially by up-regulated miR-135b-5p and miR-499a-3p in hUC-MSC-exos. The transported miR-21 from hUC-MSC-exos could inhibit PTEN expression to increase corneal epithelial wound repair [68]. This study emphasized a novel exosomal PTEN/PI3K/AKT pathway in improving corneal epithelium integrity. Similarly, Xiu et al. also verified that exosomes from miR-150-5p-overexpressing hUC-MSCs promoted skin wound healing by activating the PI3K/AKT pathway through PTEN [69]. Zhang et al. reported that miR-21-5p and miR-125b-5p, which were highly expressed in human umbilical cord blood MSC-derived exosomes (hUCB-MSC-exos), were key participants in anti-myofibroblast differentiation via TGFBR1/2 inhibition [70]. This study identified a novel strategy to reduce scar formation during the wound healing process.

### Protein from hUC-MSC-exos

Shi et al. reported that 3,3'-diindolylmethane (DIM)-induced hUC-MSCs exhibited a superior ability to promote healing of deep second-degree burn injury [71]. Furthermore, these researchers emphasized that the stemness of hUC-MSCs was significantly promoted by DIM by strengthening exosomal Wnt11/β-catenin autocrine signaling.

## WJ-MSCs

Compared with BMSCs and ADSCs, Wharton Jelly-derived MSCs (WJ-MSCs) from the umbilical cord possess excellent proliferation and pluripotency. WJ-MSCs are notable treatments for tissue injury of the spinal cord and heart, regulation of immune-related diseases, and multidirectional differentiation for tissue engineering applications [72]. In an in vitro exploration, exosomes from WJ-MSCs exhibited excellent antileishmanial and wound regenerative functions in combination with aloe-emodin [73].

## EPSCs

Epidermal stem cells (EPSCs) distributed in the basal layer of the epidermis and hair follicle protrusions are the key multipotent cells for epidermal formation, differentiation, homeostasis maintenance, re-epithelialization, and epidermal regeneration [74]. The number, proliferation, and differentiation behaviors of EPSCs are important in wound repair and regeneration. The strong paracrine ability represented by exosomes is an important mechanism by which EPSCs promote skin healing.

EPSC-derived exosomes (EPSC-exos) had outstanding efficiency in accelerating diabetic wound healing in db/db mice by inducing M2 macrophage polarization, inhibiting inflammation, promoting angiogenesis, and promoting cell proliferation in vivo, as well as promoting the activity of diabetic fibroblasts and macrophages in vitro [75]. Compared with fibroblast exosomes, EPSC-exos possessed significantly higher proportions of miRNAs. Accordingly, EPSC-exos led to the activation of TGF-β and protein kinase B signaling pathways to modulate homeostatic processes and cell differentiation. Duan et al. found that specific miRNAs were enriched from EPSC-exos in promoting wound healing, including miR-16, let-7a, miR-425-5p, and miR-142-3p [76]. In particular, miR-425-5p and miR-142-3p could inhibit myofibroblast differentiation via TGF-β1 inhibition in dermal fibroblasts. This effect was precise because of the characteristics of EPSC-exos and the loaded miRNAs. Therefore, EPSC-exos promote regenerative angiogenesis and immune regulation during wound healing.

## FD-MSCs

In regenerative medicine, fetal dermal MSCs (FD-MSCs) are novel multipotent MSCs isolated from fetal skin. FD-MSCs could resist D-galactose-induced senescence of adult dermal fibroblasts (ADFs) through paracrine effects [77]. Specifically, Wang et al. showed that FD-MSC-derived exosomes (FD-MSC-exos) activated the proliferation, migration, and secretion functions of ADFs through the Notch signaling pathway and enhanced the efficiency of wound healing [78].

## OMLP-PCs

Oral mucosa lamina propria-progenitor cells (OMLP-PCs) possess unique properties, such as easy isolation, immunosuppression, and multipotency. OMLP-PC line (OMLP-PCL) exosomes were capable of decreasing myofibroblast formation and enhancing wound repopulation [79]. OMLP-PCL exosomes also achieved scar-free healing in a preclinical wound mouse model. Manipulation of OMLP-PCL exosomes may be an optional tool for nonscarring wound healing.

## hAMSCs

Easily accessible human amnion MSCs (hAMSCs) are valuable alternative resources for nonimmunogenic and nontumorigenic stem cells with multipotential differentiation. hAMSCs are derived from the amniotic membrane of the human term placenta and are considered favorable multipotent cells with superior paracrine capabilities for obesity resistance, cartilage repair, and vascularized tissue regeneration. Gao et al. proposed that exosomal miR-135a derived from hAMSCs could directly suppress LATS2 expression, leading to superior cutaneous wound regeneration and fibroblast migration [80].

## hAFSCs

hAFSCs play an important role in wound healing and have the potential for self-renewal and easy large-scale expansion [81]. hAFSCs are multipotent immature progenitor cells obtained from human amniotic fluid. hAFSCs are competent in differentiating into various lineages, including cell types of the three embryonic germ layers, and harbor immunoregulatory properties [82]. Specific miRNAs, including let-7-5p, miR-22-3p, miR-27a-3p, miR-21-5p, and miR-23a-3p, from hAFSC-derived exosomes (hAFSC-exos) could suppress myofibroblast differentiation via TGF-β inhibition [83]. In vivo, hAFSC-exos could restrain fibrotic scar formation and promote vessel regeneration and wound healing in rat models. Exosomal miRNA-146a-5p from hAFSCs could target and inhibit CXCR4, resulting in enhanced ECM reconstruction, equilibrated collagen synthesis, increased immune-responsive molecules, and decreased inflammatory cytokines [84]. These findings proved the role of exosomal miRNA-146a-5p of hAFSCs in achieving effective anti-scarring healing and skin regeneration. Therefore, hAFSC-exos are a potential therapeutic strategy for preventing fibrotic scar formation.

## USCs

Human urine-derived stem cells (USCs) exhibit classic MSC characteristics, such as multidirectional differentiation, immune regulation, and enhanced angiogenesis [85]. Considering the advantages of feasible, safe, noninvasive, and inexpensive acquisition methods and stable culture systems, USCs are emerging as an excellent source for exosome extraction. Notably, exosomes originating from human USC-derived exosomes (USC-exos) comprised abundant wound healing-promoting proteins [85]. Among them, DMBT1 protein transfer was a crucial event for the alleviated angiogenic formation of ECs and diabetic wound healing.

## MenSCs

As a newly discovered MSC type, menstrual blood-derived MSCs (MenSCs), reported in 2007, have abundant sources, noninvasive harvesting processes, a high proliferation rate, low immune rejection, and pluripotency. MenSCs have also been assessed for their therapeutic potential in multiple sclerosis, Duchenne’s muscular dystrophy, wounds, advanced peripheral arterial disease, and congestive heart failure [86, 87]. Notably, Dalirfardouei et al. found that exosomes released from MenSCs exhibited excellent therapeutic effects on the wound healing process in a diabetic mouse model [88]. The referred mechanisms of MenSCs within this process included alleviated inflammation with M1-M2 macrophage polarization, increased re-epithelialization with NF-κB p65 subunit up-regulation and NF-κB activation, reduced scar formation, and a decreased collagen I/collagen III ratio.

## iPSC-MSCs

Induced pluripotent stem cells (iPSCs) are pluripotent stem cells that are usually produced from somatic cells by a defined transcription factor panel [89]. The superb differentiation ability of iPSCs has revolutionized the progression of drug screening, disease modeling, disease treatment, and many other fields. Notably, human iPSC-derived MSCs (iPSC-MSCs) have remarkably powerful regulatory capabilities in cellular activity, immune regulation, cytokine profile, and microenvironmental regulation.

Exosomes originating from iPSC-MSCs are a promising option for skin rejuvenation and regeneration that combine the advantages of MSCs and iPSCs without immunogenicity. Zhang et al. found that iPSC-MSC-derived exosomes (iPSC-MSC-exos) had a significant regenerative effect after subcutaneous injection around wounds in rats, accelerating re-epithelialization, reducing scar extension, and promoting collagen maturation and newly formed blood vessels. These combined effects promoted the healing of cutaneous wound outcomes [90]. Kim et al. found that both MSC-exos and iPSC-MSC-exos increased the proliferative activity and enhanced collagen secretion of HaCaT cells and HDFs [91]. This study proved that iPSC-MSC-exos accelerated skin cell proliferation by stimulating ERK1/2 phosphorylation.

## iPSC-KCs

KCs, the predominant cell type in the skin, spatially occupy the most basal and superficial layers of the stratified epithelium, serving as a defensive front for innate immunity following skin injury [92]. During the four phases of wound healing, KCs sense different microenvironmental cues and accordingly alter their migration, proliferation, differentiation capability, immune function, and pigmentation regulation [93]. Wound repair requires a substantial supply of highly proliferative KCs for support, and iPSC-derived KCs (iPSC-KCs) can be an abundant source of cells. Bo et al. found that iPSC-KC-exosomes (iPSC-KC-exos) could promote wound healing in a deep second-degree burn model in vivo with enhanced angiogenesis and re-epithelialization and promote EC and KC migration in vitro [94]. Specifically, the abundant miR-762 in iPSC-KC-exos targeted ITGB1 to modulate cell migration. Therefore, iPSC-KC-exos might serve as novel cell-free formulations for deep second-degree burn wounds.

## hESCs

Human embryonic stem cells (hESCs) were obtained from in vitro fertilized embryos. hESCs are primitive pluripotent stem cells with the capacity for self-renewal and differentiation into all somatic cells in vitro as seed cells [95]. Chen et al. reported that hESC-exos promoted pressure ulcer healing by rejuvenating senescent ECs by activating Nrf2 [96]. In addition, miR-200a was highly expressed in hESC-exos and down-regulated Keap1 to activate Nrf2 expression. hESC-exos might be an ideal nanoplatform for vascular regeneration**.**

## Discussion

**Fig. 3:**
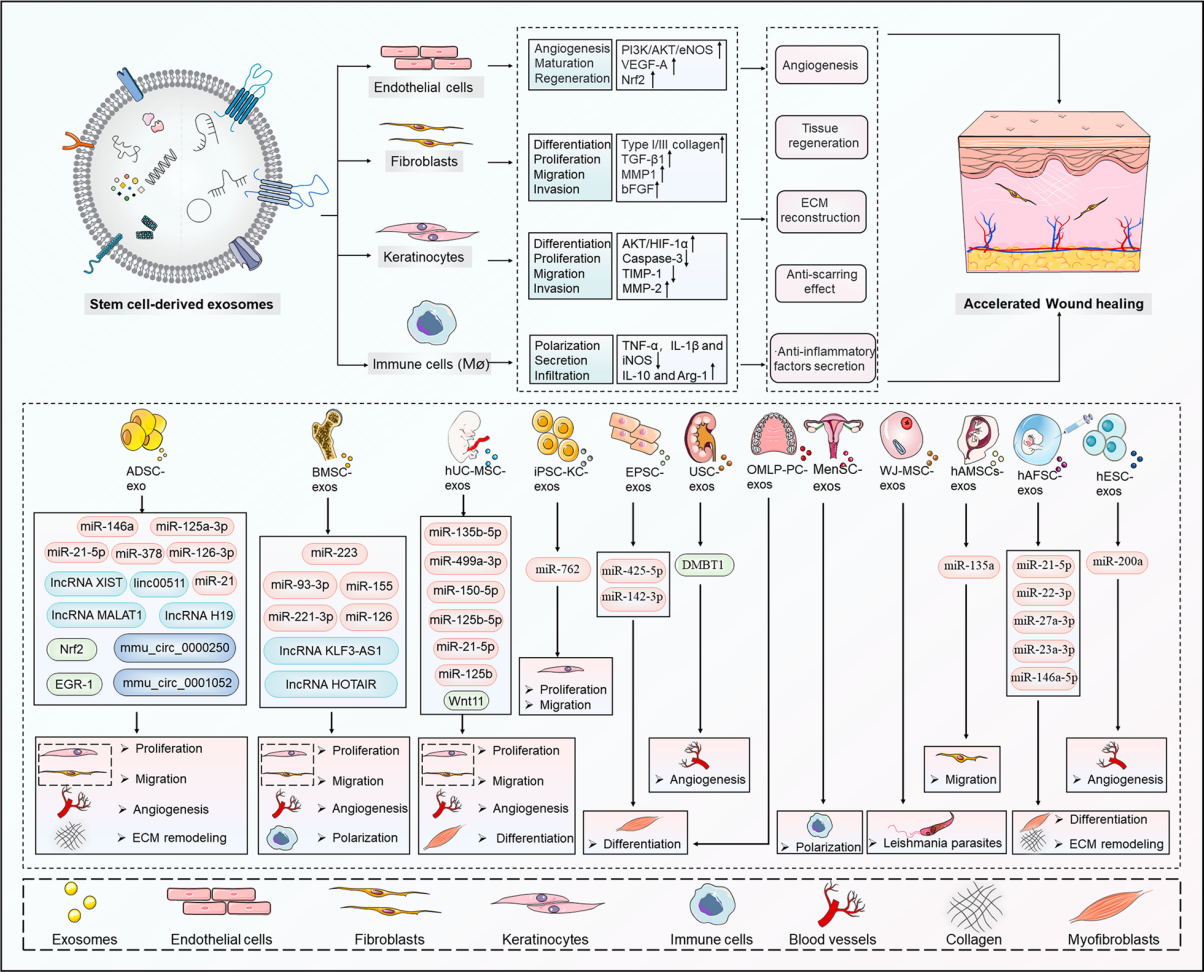
The roles and mechanisms of various MSC-exos in promoting wound healing. MSC-exos, represented by ADSCs, BMSCs, hUC-MSCs, and other stem cell types, play a role in shaping the activity of fibroblasts, KCs, immune cells, and ECs. Specifically, the uploaded and shuttled proteins and ncRNAs within MSC-exos can trigger behavioral alterations in skin cells, such as proliferation, migration, invasion, differentiation, and polarization, consequently leading to angiogenesis, anti-inflammatory factor secretion, ECM reconstruction, anti-scarring effects, and tissue regeneration. Ultimately, these effects synergistically contribute to the accelerated or anti-scarring wound healing process. Figure 3 is partly generated using Servier Medical Art (https://smart.servier.com), provided by Servier, licensed under a Creative Commons Attribution 3.0 unported license (https://creastivecommons.org/licenses/by/3.0/)

Overall, MSC-exos are widely involved in the processes of cellular activity, inflammation and immune regulation, angiogenesis, tissue fibrosis, and ECM reconstruction through the delivery of multiple bioactive molecules, opening up innovative pathways for stem cells to repair wounds in a noncellular manner **(**Fig. 3**, **Table 1**)**. However, there are still many noteworthy issues that need careful consideration, mainly MSC-exo acquisition, the complexity of mechanisms, carrier characteristics, and clinical applications [97, 98].

**Table 1:** The expression patterns, mechanisms, and clinical values of MSC-exos in wound healing

Exosomal substance	Sample source	Expression	Mechanisms	Clinical values	References
miR-21-5p	miR-21-5p-modified ADSC-exos	Overexpression	Promoted proliferation and migration of KCs via Wnt/β-catenin signaling	A cell-free therapy for wound healing treatment	[28]
miR-146a	miR-146a-modified ADSC-exos	Overexpression	Promoted the mobility and proliferation of fibroblasts with up-regulation of SERPINH1 and p-ERK	A novel target for wound healing	[29]
miR-378	ADSC-exos	Up-regulation	Decreased oxidative stress injury in HaCaT cells by targeting caspase-3	A protective strategy in wound healing	[30]
miR-125a-3p	miR-125a-3p-modified ADSC-exos	Overexpression	Promoted angiogenesis of wound healing via PTEN inhibition	A specific thought for the remedy of tissue repair	[31]
miR-126-3p	miR-126-3p-modified ADSC-exos	Overexpression	Increased collagen deposition and angiogenesis by inhibiting PIK3R2	A new therapeutic target for skin reconstruction	[33]
miR-21	ADSC-exos; miR-21-modified hUC-MSC-exos	Up-regulation; overexpression	Promoted MMP-9 and inhibit TIMP-2 by activating PI3K/AKT to promote proliferation and migration of HaCaT; promoted corneal EC proliferation and migration by up-regulating PI3K/AKT/PTEN	A potential target for promoting wound healing and corneal epithelium integrity	[34, 61]
linc00511	linc00511-modified ADSC-exos	Overexpression	Facilitated angiogenesis by inhibiting PAQR3-induced degradation of Twist1 ubiquitination	A basis for promoting DFU healing	[35]
lncRNA H19	lncRNA H19-modified ADSC-exos and BMSC-exos	Overexpression	Up-regulated SOX9 expression via miR-19b to activate Wnt/β-catenin to promote proliferation, migration and invasion of fibroblasts; prevented apoptosis and inflammation of fibroblasts by inhibiting miR-152-3p/PTEN axis	A potential target for accelerating wound healing, including DFU	[36, 56]
lncRNA XIST	ADSC-exos	Up-regulation	Facilitated fibroblast proliferation and migration via suppressing miR-96-5p and promoted DDR2 expression	A perfect target for wound healing	[37]
lncRNA MALAT1	ADSC-exos	Up-regulation	Promoted HaCaT and fibroblast proliferation, migration by activating Wnt/β-catenin via binding miR-124 and inhibited apoptosis via miR-378a/FGF2	A therapeutic target for promoting cutaneous wound healing	[39, 40]
mmu_circ_0000250	mmu_circ_0000250-modified ADSC-exos	Overexpression	Increased angiopoiesis through enhancing SIRT1 expression by miR-128-3p absorption	A target for diabetic wound healing	[41]
mmu_circ_0001052	mmu_circ_0001052-modified ADSC-exos	Overexpression	Promoted angiogenesis through miR-106a-5p and FGF4/p38MAPK pathway	A potential target of DFU treatment	[42]
Nrf2	Nrf2-modified ADSC-exos	Overexpression	Suppressed ROS and inflammatory cytokine expression	Facilitating DFU wound healing	[43]
EGR-1	ADSC-exos	Up-regulation	Increased angiogenesis by regulating EGR-1/lncRNA-SENCR/DKC1/VEGF-A axis	Accelerating wound healing process	[44]
miR-126	DFO-pretreated BMSC-exos; miR-126-modified BMSC-exos	Up-regulation; overexpression	Enhanced angiogenesis via down-regulating PTEN expression to activate the PI3K/AKT pathway; promoted HUVEC angiogenesis by targeting PIK3R2/PI3K/AKT pathway	Promoting normal and diabetic wound healing	[46, 52]
miR-221-3p	ATV-pretreated BMSC-exos	Up-regulation	Enhanced angiogenesis by stimulating the activation of AKT/eNOS pathway	A promising treatment for wound closure	[47]
miR-93-3p	BMSC-exos	Up-regulation	Promoted migration of HaCaT cells by targeting miR-93-3p/APAF1 axis	A promising target for wound healing	[50]
miR-155	miR-155-inhibitor-loaded BMSC-exos	Down-regulation	Suppressed FGF-7 and promoted inflammatory effect	Promoting diabetic wound healing	[51]
miR-223	miR-223-modified BMSC-exos	Overexpression	Modulated the M2 polarization phenotype by targeting pknox1	A potential target for wound healing	[53]
lncRNA KLF3-AS1	lncRNA KLF3-AS1-modified BMSC-exos	Overexpression	Boosted angiogenesis by down-regulating miR-383	Promoting diabetic skin wound healing	[54]
lncRNA HOTAIR	lncRNA HOTAIR-modified BMSC-exos	Overexpression	Enhanced fibroblasts proliferation and migration	A new target toward diabetic wound repair	[55]
agomiR-125b	hUC-MSC-exos	Overexpression	Alleviated hypoxia-induced cell apoptosis mediated by exosomal miR-125b/TP53INP1	A powerful strategy for promoting skin injury	[59]
miR-135b-5p, miR-499a-3p	455-nm blue light exposure hUC-MSC-exos	Up-regulation	Increased angiogenic ability via stimulating ECs	A potent tool for tissue repair and regeneration	[60]
miR-150-5p	miR-150-5p-modified hUC-MSC-exos	Overexpression	Promoted proliferation and migration of HaCaT cells via activating PI3K/AKT pathway through PTEN	A novel strategy to improve tissue wound healing	[62]
miR-21-5p, miR-125b-5p	miR-21-5p-modified hUCB-MSC-exos, miR-125b-5p-modified hUCB-MSC-exos	Overexpression	Inhibited myofibroblast differentiation via the TGF-β signaling pathway	A strategy to prevent scar formation during wound healing process	[63]
Wnt11	DIM-induced hUC-MSC-exos	Overexpression	Increased collagen I/III expression via Wnt11/β-catenin autocrine signaling activation	For healing of deep second-degree burn injury	[64]
miR-762	iPSC-KC-exos	Up-regulation	Targeted ITGB1 to modulate EC and KC migration	For deep second-degree burns	[70]
agomiR-425-5p, agomiR-142-3p	EPSC-exos	Overexpression	Inhibited myofibroblasts differentiation via TGF-β1 inhibition	Reducing scar in wound healing process	[73]
DMBT1	USC-exos	Up-regulation	Promoted angiogenic effect	Promoting diabetic wound healing	[76]
miR-135a	miR-135a-modified hAMSC-exos	Overexpression	Suppressed LATS2 levels to increase fibroblast migration	A superior candidate for cutaneous wound regeneration	[83]
let-7-5p, miR-21-5p, miR-22-3p, miR-27a-3p, miR-23a-3p	hAFSC-exos	Up-regulation	Suppressed myofibroblast differentiation via inhibiting TGF-β	Hair follicle regeneration and anti-scarring treatment	[84]
miR-146a-5p	hAFSC-exos	Up-regulation	Promoted ECM remodeling by down-regulating CXCR4 and SDF1	Promoting anti-scarring healing	[85]
miR-200a	hESC-exos	Up-regulation	Down-regulated Keap1 to activate Nrf2 expression to rejuvenate senescent ECs	For pressure ulcer healing	[87]

*KCs* keratinocytes, *ECs* endothelial cells, *DFU* diabetic foot ulcer, *DFO* deferoxamine, *HUVECs* human umbilical vein endothelial cells, *ATV* atorvastatin, *DIM* 3,3′-diindolylmethane, *ITGB1* integrin subunit beta 1, *ECM *extracellular matrix

First, exosomes have limitations in terms of origin, isolation, purification, and identification. Clinical application of MSC-exos necessitates rigorous quality management, thus requiring a high degree of standardization involving the isolation of cells, culture serum, and exosomes [99, 100]. The techniques for exosome isolation are diverse and include ultracentrifugation, size filtration, size exclusion chromatography, polymer precipitation, and several emerging combined techniques. However, there is no specific ideal method to rapidly and efficiently obtain MSC-exos. Currently, there is no fully standardized procedure for the isolation, transport, and preservation of MSC-exos or for their identification, which involves a comprehensive characterization of exosome contents and biomarkers.

MSCs are characterized by a distinct ability to expand in vitro, but this does not compensate for the low proportion of cells in their niches. Moreover, for clinical applications, it is a major challenge to achieve large amounts of quality-controlled MSC-exos [101]. To address this issue, researchers should consider clinical needs in terms of expanding MSC culture, large-scale exosome expansion, and exosome isolation/purification/quality control [102]. Isolated MSCs must undergo effective in vitro expansion to obtain a large number of high-quality MSCs in a rapid period of time for exosome extraction. As the content of MSC-exos is not static, exosome abundance is determined by MSC tissue origin, MSC differentiation and proliferative activity, and culture conditions. Therefore, the therapeutic efficiency of MSCs could be improved by a suitable source of MSCs and optimized activity. Second, the efficiency of MSC-exo expansion is improved depending on different culture factors, including basal medium, glucose concentration, stable glutamine, MSC passaging, and inducers of relevant proteins in exosome biogenesis [103]. In addition, the traditional two-dimensional expansion of MSCs has difficulty obtaining sufficient cell volume, and too many passages can cause a decrease in MSC quality. A three-dimensional culture approach may help to increase the abundance of stem cells and MSC-exos obtained [104]. Therefore, improved protocols and standardized procedures are needed to achieve a balance of increased MSC-exo yield and purity.

Second, MSC-exo components are rather sophisticated, containing a variety of genes, proteins, and metabolic molecules, while wound healing involves multiple cell types and the complexity of spatiotemporal changes [105]. Most previous studies have focused on the analysis of the role of single components of MSC-exosomes and their effects on specific skin cells, but a systematic assessment of the active components of exosomes and their effects on multiple skin cells is lacking. In other words, since wound healing is regulated by multiple levels, factors, and cells, the effect of MSC-exos on wound healing is the result of a combination of these factors [106]. The validation of the effectiveness of a single component in MSC-exos is a partial, not a comprehensive, consequence. Moreover, the wound healing efficacy of MSC-exos can be greatly enhanced by overexpressing specific molecules of MSC-exos.

Third, MSC-exos can be used as endogenous carriers, which have lower immunogenicity and avoid phagocytosis of the reticuloendothelial system compared to exogenous nanocarriers [107]. Based on their endogenous, biocompatible, and multifunctional properties, exosomes are becoming a new tool for drug delivery systems, immunotherapy, and precision therapy. Wound healing materials with specific components and spatial structures can provide an optimal physical microenvironment, such as humidity, for wound repair and regulate the biological functions of skin cells to improve the speed and quality of wound repair. The combination of wound repair materials and MSC-exos is also a promising research direction to promote wound repair and regeneration [108]. For instance, MSC-exos stimulated with Fe3O4 nanoparticles (mag-BMSC-exos) and a static magnetic field synergistically promoted wound regeneration by up-regulating miR-21-5p expression [109]. Our main focus here is on the biological properties of exosomes, while we have not explored the materials piggybacking on exosomes in depth.

Last, wound healing and skin regeneration are complex physiological processes that require the synergistic action of multiple tissues and cells to replace, repair, and reconstruct the missing cellular structures and tissue levels [110]. Most studies have focused on the cellular and animal level and are at the preclinical stage, while there are almost no reports of clinical applications of MSC-exos in wound repair. The repair environment of animal model wounds does not realistically reproduce the complexity and realism of clinical wounds. In addition, keloids do not grow in the mouse wound model, which is very different from keloid formation after human wounds [111]. Moreover, there are many types of wounds involved, including common wounds, diabetic wounds, eczema wounds, and tumor wounds [112]. The types of wounds for which different stem cells are suitable may be different, and this has not been studied. In particular, whether the activity of stem cells in tumor wounds has any influence on the growth and recurrence of tumors needs to be seriously considered. Therefore, there are still many open questions regarding the clinical application of exosomes, such as the source of cells, therapeutic dose, frequency of administration, and effect on keloid formation. For example, the combined therapy of BMSC-exos with the small molecule Nrf2 activator tert-butylhydroquinone (tBHQ) might collaboratively improve wound healing with a higher efficiency [113]. Therefore, combining different types of MSC-exos with conventional treatment modalities to promote wound healing is also a promising therapeutic strategy. Ongoing basic research and clinical trials to elucidate the mechanism of activity, efficacy, and safety of MSC-exos in depth are extremely urgent.

## Conclusion

Overall, various MSC-exos, mainly derived from ADSCs, BMSCs, hUC-MSCs, iPSCs, EPSCs, FD-MSCs, USCs, OMLP-PCs, MenSCs, WJ-MSCs, hAMSCs, hAFSCs, and hESCs, play a role in promoting the activity of fibroblasts, KCs, immune cells, and ECs in diabetic wounds, inflammatory wound repair, and even wound-related keloid formation. Exosomal ncRNAs and proteins are the most reported MSC-exo contents that can affect phenotypic changes in skin-associated cells. Deciphering the biological properties of MSC-exos as cell-free therapeutic tools is important for wound healing and cutaneous regeneration.

## Data Availability

Not applicable.

## Abbreviations

DIM: 3,3′-Diindolylmethane
ADSCs: Adipose-derived stem cells
ADSC-exos: ADSC-derived exosomes
ATV: Atorvastatin
ATV-exos: Atorvastatin-pretreated BMSC-exos
BMSC-exos: BMSC-derived exosomes
PGZ-exos: BMSC-exos pretreated with pioglitazone
BMSCs: Bone marrow-derived MSCs
circRNAs: Circular RNAs
DFO-exos: Deferoxamine-pretreated BMSC-exos
DFU: Diabetic foot ulcer
ECs: Endothelial cells
EPCs: Endothelial progenitor cells
EPSCs: Epidermal stem cells
EPSC-exos: EPSC-derived exosomes
ECM: Extracellular matrix
FD-MSC-exos: FD-MSC-derived exosomes
FD-MSCs: Fetal dermal MSCs
hAFSC-exos: HAFSC-derived exosomes
HGF: Hepatocyte growth factor
hESC-exos: HESC-derived exosomes
HG: High glucose
hUC-MSC-exos: HUC-MSC-derived exosomes
hAFSCs: Human amniotic fluid stem cells
hAMSCs: Human amniotic membrane MSCs
HDFs: Human dermal fibroblasts
hESCs: Human embryonic stem cells
HSFs: Human skin fibroblasts
hUCB-MSC-exos: Human umbilical cord blood MSC-derived exosomes
hUC-MSCs: Human umbilical cord MSCs
HUVECs: Human umbilical vein ECs
iPSCs: Induced pluripotent stem cells
IL-10: Interleukin-10
IL-6: Interleukin-6
iPSC-KCs: IPSC-derived keratinocytes
iPSC-MSCs: IPSC-derived MSCs
iPSC-KC-exos: IPSC-KC-exosomes
iPSC-MSC-exos: IPSC-MSC-derived exosomes
KCs: Keratinocytes
lncRNAs: Long non-coding RNAs
MT-exos: Melatonin-pretreated exosomes
MenSCs: Menstrual blood-derived MSCs
MSCs: Mesenchymal stem cells
miRNAs: MicroRNAs
MSC-exos: MSC-derived exosomes
mag-BMSC-exos: MSC-exos with the stimulation of Fe3O4 nanoparticles
NGFs: Nerve growth factors
ncRNAs: Non-coding RNAs
OMLP-PCL: OMLP-PC line
OMLP-PCs: Oral mucosa lamina propria-progenitor cells
PI3K: Phosphoinositide-3 kinases
PCNA: Proliferating cell nuclear antigen
ROS: Reactive oxygen species
tBHQ: Tert-butylhydroquinone
TGF-β1: Transforming growth factor-beta 1
USCs: Urine-derived stem cells
USC-exos: USC-derived exosomes
VEGF: Vascular endothelial growth factor
WJ-MSCs: Wharton Jelly-derived mesenchymal stem cells
